# Morphological and Transcriptomic Analyses of the Adrenal Gland in *Acomys cahirinus*: A Novel Model for Murine Adrenal Physiology

**DOI:** 10.3390/cells14181431

**Published:** 2025-09-12

**Authors:** Alina Bilyalova, Airat Bilyalov, Olga Kozlova, Nikita Filatov, Daria Filimoshina, Guzel Gazizova, Ruslan Deviatiiarov, Angelina Titova, Andrey Bydanov, Yana Mukhamedshina, Elena Shagimardanova, Andrey Kiyasov, Dmitry Tychinin, Mary Woroncow, Oleg Gusev

**Affiliations:** 1 Institute of Fundamental Medicine and Biology, Kazan Federal University, 420008 Kazan, Russia; 2 National Medical Research Centre of Cardiology Named After Academician E.I. Chazov, 121552 Moscow, Russia; 3 Loginov Moscow Clinical Scientific Center, 111123 Moscow, Russia; 4 Life Improvement by Future Technologies (LIFT) Center, 121205 Moscow, Russia; 5 Intractable Disease Research Center, Graduate School of Medicine, Juntendo University, Tokyo 113-8421, Japan; 6 Endocrinology Research Centre, Institute of Personalized Medicine, 117292 Moscow, Russia; 7 Genomics and Bioimaging Core Facility, Skolkovo Institute of Science and Technology, 143026 Moscow, Russia; 8 Federal State Budgetary Institution «Centre for Strategic Planning and Management of Biomedical Health Risks» of the Federal Medical and Biological Agency (Centre for Strategic Planning, of the Federal Medical and Biological Agency), 119121 Moscow, Russia; 9 Faculty of Medicine, Medical Research and Education Institute, Lomonosov Moscow State University, 119991 Moscow, Russia

**Keywords:** *Acomys cahirinus*, spiny mouse, adrenal cortex, steroidogenesis, animal mode, *CYP17a1*

## Abstract

This study investigates the adrenal gland structure and gene expression in *Acomys cahirinus* compared to *Mus musculus*, aiming to assess its relevance for human adrenal disease modeling. We identified a well-defined zona reticularis in *Acomys*, resembling the human adrenal cortex. Transcriptomic analysis revealed upregulation of key steroidogenic genes (C*yp17a1*, *Sult1e1*, *Hsd3b2*, *Defb18*, *Kiss1*, *H2-Ke6*), with validation by qRT-PCR and CAGE-seq. The gene *Nupl1* showed discordant results between RNA-seq and CAGE. Pathway analysis highlighted enrichment of steroidogenesis and adrenal metabolism. Notably, a GTEx-based comparison demonstrated that *Acomys* adrenal gene expression closely mirrors the expression in the human adrenal cortex, whereas *Mus musculus* samples diverged toward brain-specific signatures. These findings suggest that *Acomys cahirinus* represents a promising model for adrenal research, though further studies including single-cell transcriptomics and functional assays are warranted to fully establish its translational potential.

## 1. Introduction

The human adrenal cortex is structured into three concentric layers, recognized as the zona glomerulosa (ZG), zona fasciculata (ZF), and zona reticularis (ZR) [1]. Aldosterone is a mineralocorticoid hormone synthesized in the adrenal cortex. This hormone regulates electrolyte levels by balancing the potassium and sodium concentrations in the blood [2]. The zona fasciculata is located under the zona glomerulosa and is responsible for glucocorticoid synthesis, while minor secretion of androgens is concurrently carried out [3]. The innermost layer of the adrenal cortex, the zona reticularis, responsible for the synthesis of androgens with low biological activity, including dehydroepiandrosterone (DHEA), dehydroepiandrosterone sulfate (DHEA-S), androstenedione, androstenediol, 11β-hydroxyandrostenedione, and small amounts of glucocorticoids, is also present [1].

A widely utilized standard in the experimental study of adrenal diseases is animal models, which employ rodents such as mice and rats [4]. However, these animals have specific adrenal cortex structures without obvious homology in humans [5]. For instance, mice and rats lack the zona reticularis responsible for androgen production. In rodents, a morphologically distinct ZR is not consistently identifiable: in adult mice, it is typically absent, and in rats, its delineation is context-dependent and influenced by the presence of the undifferentiated zone [6]. Functionally, adult mice and rats lack adrenocortical *CYP17A1* and therefore do not produce adrenal androgens, in contrast to species with a well-defined, androgen-producing ZR [7]. Consequently, the predominant glucocorticoid in rodents is corticosterone, whereas in humans, it is cortisol [8]. This enzyme also plays a key role in the adrenal biosynthesis of androgens in humans. At the same time, rodents lack this enzymatic activity in their adrenal glands, which makes them unable to produce androgens endogenously in the adrenal cortex [8].

The advancement of gene and cellular treatment approaches for various adrenal diseases requires preclinical investigations involving laboratory animals. Conventionally, laboratory rodents such as mice and rats are used for these studies. However, a pertinent query arises: is their utilization as models for studying adrenal diseases and, moreover, for assessing novel therapies truly justified?

Recently, there has been a growing interest in employing mice of the genus *Acomys* as novel animal models to study enhanced regenerative capabilities. Spiny mice are the only mammals that are capable of regenerating tissues without fibrosis [9,10,11,12,13]. Additionally, Quinn et al. demonstrated that the adrenal gland of spiny mice can synthesize and secrete DHEA from at least 30 days of gestation, comparable to the human fetus. Their study, which involved dexamethasone treatment and gestational sampling, revealed that spiny mouse adrenals express the active enzyme *CYP17A1* in ZR, crucial for cortisol production [5]. Notably, this research employed *Acomys* as a genus without specifying a particular species. In particular, cortisol and aldosterone were both detected in fetal plasma [14].

This noteworthy discovery opens up the possibility that the spiny mouse may represent a more relevant model for studying adrenal diseases. The primary objective of this research was to compare the morphological structure and gene expression level of the adrenal glands between Balb/c mice (a standard laboratory animal) and one of the species of mice belonging to spiny mice—*Acomys cahirinus*.

## 2. Materials and Methods

### 2.1. Animals and Ethical Issues

The experimental procedures were conducted on sexually mature male *Acomys cahirinus* mice (n = 6) and Balb/c mice (n = 6) at 11–12 weeks of age. Ethical approval for all the animal studies was obtained from the local Ethics Committee of the Institute of Fundamental Medicine and Biology, Kazan (Volga region) Federal University, under extract No. 40, dated 9 March 2023.

Zoletil 100 was administered as an analgesic at a dosage of 7 mg per kilogram of body weight. Subsequently, the anterior abdominal wall was depilated, and the animal was securely affixed to the surgical table. Surgical access was established in the left lateral region by making an incision in the anterior abdominal wall, positioned 1 cm to the left of the anterior midline. The excised adrenal gland tissues were subsequently earmarked for subsequent morphological investigations and for the analysis of gene expression. The animals were euthanized by decapitation under deep anesthesia induced with Zoletil 100.

### 2.2. Histological Procedure

Obtained tissue fragments were fixed in 10% neutral formalin in 0.2 M phosphate buffer (pH = 7.4) for 24 h. After fixation, the tissues were processed for paraffin embedding using an automated tissue processor (STP 420 ES, Thermo Scientific, Waltham, MA, USA). The protocol included sequential dehydration in four changes of isopropanol with increasing concentration, followed by two changes of an intermediate isopropanol–mineral oil mixture (3 h total) and then transfer to pure mineral oil. Paraffin infiltration was performed in three changes of paraffin, each for 1 h, after which the tissues were embedded in paraffin blocks. The paraffin blocks were sectioned at 4 µm thickness using a rotary microtome (Microm HM 366S, Thermo Scientific, Waltham, MA, USA). The tissue preparations were stained using Mallory (Ergoprodaction, Biovitrum, Russia) and specific antibodies against tyrosine hydroxylase (Th) (AF6113, Affinity Biosciences, Jiangsu province, China, 1:100), steroidogenic acute regulatory protein (StAR) (DF6192, Affinity Biosciences, Jiangsu province, China, 1:100), HSD3B2 (DF6639, Affinity Biosciences, Jiangsu province, China, 1:100), and Cytochrome P450 17A1 (DF3563, Affinity Biosciences, Jiangsu province, China, 1:100).

Digital histological images were acquired using the NanoZoomer S60 Digital slide scanner (Hamamatsu Photonics, Hamamatsu, Japan). The NDP program.view2 (Hamamatsu, Japan) was utilized for the comprehensive analysis of the digital histological images.

### 2.3. Immunofluorescence Analysis

Immunofluorescence reactions were conducted in a standard way. The sections were incubated with primary and secondary antibodies to identify the antigen: *CYP17A1* (Affinity Biosciences, 1:100) and donkey anti-mouse Alexa Fluor 555 (Thermo Fisher Scientific, Waltham, MA, USA, 1:200). The tissue sections were observed using an LSM 700 confocal microscope (Carl Zeiss, Oberkochen, Germany). Only the cells with nuclei clearly outlined by DAPI (10 µg/mL in PBS, Sigma, St. Louis, MO, USA) were considered. Negative controls were obtained using the same protocol but without the addition of primary or secondary antibodies.

### 2.4. RNA Sequencing and Analysis

The adrenal gland tissues were obtained from three animals in both groups. RNA was extracted according to the manufacturer’s instructions using a Qiagen RNeasy Mini Kit (Qiagen). The quality of the total RNA was evaluated using a Bioanalyzer 2100 (Agilent Technologies, Santa Clara, CA, USA). The quantity and purity of the RNA were estimated on a NanoPhotometer (Implen, Munich, Germany). A total of 800 ng of RIN ≥ 7 total RNA was used for library construction using the NEBNext^®^ Poly(A) mRNA Magnetic Isolation Module and NEBNext^®^ Ultra II™ Directional RNA Library Prep Kit for Illumina (New England Biolabs) according to the manufacturer’s instructions. The quality of the libraries was verified using the Bioanalyzer 2100 (Agilent Technologies, Santa Clara, CA, USA), and the yield was validated by qPCR. The libraries were then sequenced on a NovaSeq6000 (Illumina, San Diego, CA, USA) with pair-end 65 bp reading and consisted of approximately 42 mln read pairs per sample on average (three replicates for Balb/c and three for *Acomys*). The reads of *Acomys cahirinus* were mapped onto the genome downloaded from Elizabeth Dong Nguyen et al.’s report with the hisat2 tool (version 2.2.1) [15]. As for the Balb/c mouse, we used the mouse genome assembly version GRCm39. Hisat2 indices were prepared using files with exons and splice sites created previously from a gtf file (annotation). The mapping statistics were constant and similar between species groups (an approximately 97% overall mapping rate and approximately 88% unambiguous mapping).

The raw counts were then calculated with the htseq count tool (version 2.0.4) [16]. Differential expression analysis was carried out using the edgeR package (version 3.36.0) of R (version 4.2.2).

Gene-set enrichment: Over-representation analysis was performed with gseapy v1.0.3 using the Enrichr GTEx_Tissue_Expression_Up and BioPlanet_2019 libraries. Significance was assessed using two-sided Fisher’s exact tests followed by Benjamini–Hochberg correction; gene sets with adjusted *p* (FDR) < 0.05 were considered enriched.

### 2.5. Validation of RNA-Seq Data

Differential expression calls obtained from the bulk RNA-seq were independently validated by two orthogonal approaches.

CAGE-seq libraries were prepared from 5 mg of RIN ≥ 7 total RNA according to a previously described protocol [17]. Libraries were prepared in batches of eight and further sequenced on a NextSeq 550 System (Illumina, USA) with single-end 75 bp reading.

The sequenced CAGE reads were trimmed with the FASTX Toolkit v0.0.14. Reads with N nucleotides, matched to rRNA, or matched to adapters were removed using RNAdust v1.06 and Trimmomatic v0.38. The trimmed reads were then aligned to the mm39 genome assembly for mouse and previously mentioned assembly for *A. cahirinus* with STAR v2.7.10a. The CAGE transcription start sites, clusters, and active promoters were identified by using PromoterPipeline and TSSClassifier [18,19]. Promoter-to-gene annotation was carried out with the ChIPseeker package v1.40.0 for R, and cross-species differential expression analysis was performed as described in Alam et al. 2020 [19,20].

The gene expression results obtained by the NGS approach were validated by means of real time PCR. RT-qPCR was performed in 96-well plates using Bio-Rad CFX Real-Time PCR equipment (Bio-Rad, Hercules, CA, USA) with a One Step TB Green PrimeScript RT-PCR Kit II (Takara, Japan) according to the manufacturer’s instructions. Each reaction had a final volume of 10 μL and contained the following components: 1 μL of RNA, 5 μL of 2X One Step TB Green RT-PCR Buffer 4, 0.4 μL of PrimeScript 1 step Enzyme Mix 2, 0.4 μL of forward primer (10 μmol/L), 0.4 μL of reverse primer (10 μmol/L), and 2.3 μL of ddH_2_O. The primer sequences are shown in Supplementary Table S1.

The reaction was conducted at 42 °C for 5 min followed by 95 °C for 10 s, followed by 40 cycles of denaturation at 95 °C for 5 s, annealing at 59 °C for 30 s, and extension at 72 °C for 60 s. Each RT-qPCR involved three biological replicates and three technical replicates. Bio-Rad CFX Maestro software (Bio-Rad, Hercules, CA, USA, latest v. 2.3) was used for the calculation of the Cq values of each amplification. The GAPDH and ACTB genes were used as reference genes.

### 2.6. Statistical Analysis

Statistical analysis of the experimental results was performed using GraphPad Prism version 9.0. The normality of the distribution was assessed using the Shapiro–Wilk, D’Agostino–Pearson, and Kolmogorov–Smirnov criteria. Unpaired t-tests (parametric) were utilized to determine the statistical significance of the observed differences between the data arrays of the two groups of data. Statistical significance was considered at *p* < 0.05.

## 3. Results

### 3.1. Histological Examination

**Figure 1:**
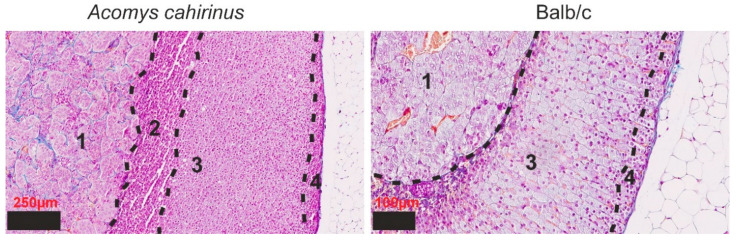
Mallory’s trichrome staining of the structure of the adrenal gland of *Acomys cahirinus* and Balb/c: 1—medulla; 2—zona reticularis; 3—zona fasciculata; 4—zona glomerulosa. The dashed lines denote the boundary between the zones of the adrenal gland.

Upon exposure to standard staining dyes, the adrenal glands of Balb/c mice exhibit discernible features of both the medulla and cortex. The medulla is marked by the presence of elongated clusters of sizable rounded cells, encased within a thin layer of connective tissue. In parallel, the cortex distinctly manifests as two separate and easily distinguishable zones: the outer layer, referred to as the zona glomerulosa, adopts a formation evocative of arched clusters comprising rounded cells, distinguished by a substantial abundance of nuclei; conversely, the inner layer, denoted as the zona fasciculata, presents an organized arrangement of cellular plates that adopt a columnar structure (Figure 1).

Within the *Acomys* group, two primary zones were identified. The medulla is similarly characterized by the presence of clusters of sizable rounded cells with high amounts of connective tissue. Morphologically, the zona glomerulosa and fasciculata within the cortex do not exhibit a distinction from their counterparts in Balb/c mice. However, an additional region was observed, comprising a substantial quantity of small cells arranged in a random manner, lacking the characteristic formations seen in arches or columns. This distinctive area is presumed to constitute the zona reticularis.

**Figure 2:**
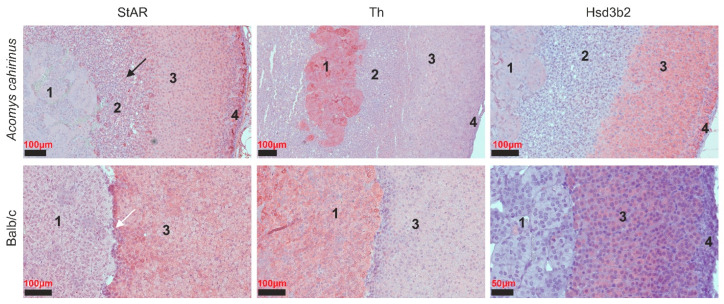
Immunohistochemistry staining of the structure of the adrenal gland of *Acomys cahirinus* and Balb/c. StAR—steroidogenic acute regulatory protein; Th—tyrosine hydroxylase; Hsd3b2-3β—hydroxysteroid dehydrogenase. The black arrow indicates the zona reticularis. The white arrow indicates the layer of cells that separates the medulla from the cortex. Labels: 1—medulla; 2—zona reticularis; 3—zona fasciculata; 4—zona glomerulosa.

We used antibodies targeting Th and StAR to distinguish the cortex and medulla (Figure 2).

To foster a more comprehensive understanding of the distinctive strengths and constraints inherent in each model, a brief elucidation is warranted of the configuration of human adrenal glands and the process of steroidogenesis before embarking on the discourse surrounding alternative animal models for the examination of congenital adrenal hyperplasia.

Tyrosine hydroxylase is an enzyme that conducts the formation of catecholamines such as epinephrine and norepinephrine. In *Acomys* and Balb/c mice, the expression of TH is observed within the adrenal medulla.

In *Acomys cahirinus*, the cortex is stained by StAR protein; however, it is notable that the medulla is located within the thickness of the zona reticularis, lacking a distinct demarcation (Figure 2). In the case of Balb/c mice, cortex staining is also evident due to StAR antibodies, yet the medulla is demarcated from the zona fasciculata of the cortex by an additional thin layer of cells that do not show nonspecific staining of StAR protein. To differentiate the zones of the adrenal cortex, we selected antibodies to the enzyme 3β-hydroxysteroid dehydrogenase (3β-HSD). This enzyme catalyzes the biosynthesis of progesterone from pregnenolone, 17α-hydroxyprogesterone from 17α-hydroxypregnenolone, and androstenedione from DHEA in the adrenal glands [21]. According to the literature, in human adrenal glands, 3β-HSD, unlike zona reticularis, is highly expressed in the zona glomerulosa and the zona fasciculata [22].

In the *Acomys* group, a positive reaction is observed in the zona glomerulosa and fasciculata, and in the zona reticularis, there is no expression of this enzyme, which is fully correlated with the data of the human adrenal gland [22]. In the group of Balb/c mice, the presence of the enzyme 3β-HSD is observed only in the cortex (zona glomerulosa and zona fasciculata) (Figure 2).

**Figure 3:**
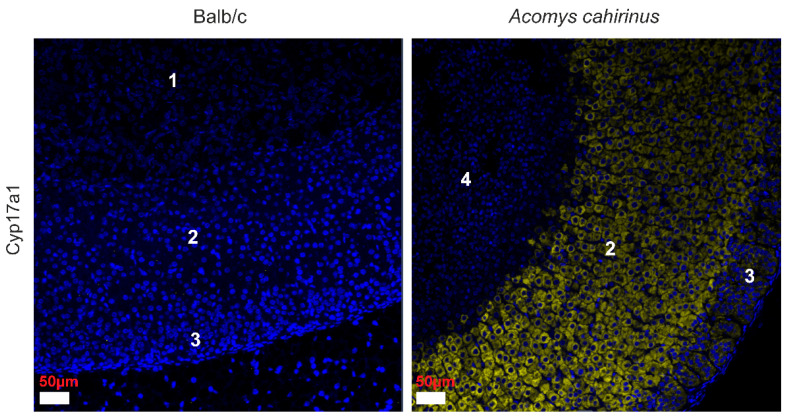
Immunofluorescence staining with *CYP17A1* (Cytochrome P450 17A1) of Balb/c and *Acomys cahirinus* adrenal gland: 1—medulla; 2—zona fasciculata; 3—zona glomerulosa; 4—zona reticularis. *CYP17A1* (P450) is shown in yellow, and nuclei are counterstained with DAPI (blue).

We employed antibodies against Cytochrome P450 17A1 to examine adrenal cortex tissue from *Acomys* and Balb/c mice. In line with Quinn’s findings, *Acomys* displayed *CYP17A1* expression in all cortical layers except the ZR, while Balb/c mice showed no detectable expression in any adrenal zone, as depicted in Figure 3.

### 3.2. Differential Gene Expression in the Adrenal Glands of Acomys Cahirinus and Balb/C Mice

**Figure 4:**
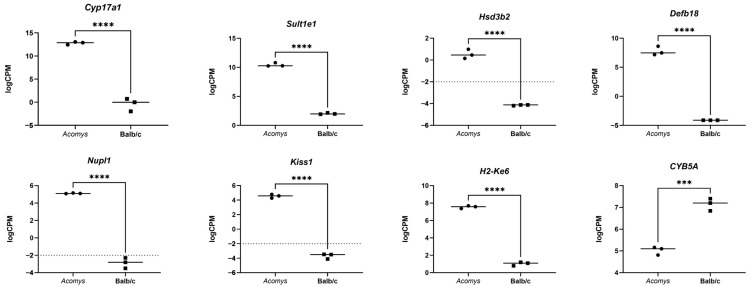
Expression of *Cyp17a1*, *Sult1e1*, *Hsd3b2*, *Defb18*, *Nupl1*, *Kiss1*, *H2-Ke6*, and *Cyb5a* genes in adrenal glands of *Acomys cahirinus* and Balb/c mice. The results are presented as mean ± SD. Statistical processing method: Unpaired *t*-test. **** *p* < 0.0001; *** *p* < 0.001. n = 3.

Using RNA sequencing, we identified differential expression gene (DEG) patterns in the adrenal glands of *Acomys cahirinus* and Balb/c mice (Figure 4). Detailed lists of differentially expressed genes and a Volcano plot are provided in Supplementary Table S2 and in Supplementary Figure S1. Notable differentially expressed genes include *Cyp17a1* (17-alpha-hydroxylase, 17,20-lyase), which is essential for cortisol and adrenal androgen synthesis [23]; *Sult1e1* (estrogen sulfotransferase), which encodes an enzyme involved in the sulfation of estrogens and other steroids, thus influencing androgen metabolism and regulation [24]; and *Hsd3b2* (3-beta-hydroxysteroid dehydrogenase), crucial for the conversion of pregnenolone to progesterone and dehydroepiandrosterone to androstenedione [25]. Furthermore, *Nme3* (nucleoside diphosphate kinase 3), *Defb18* (defensin beta 18), and *Nupl1* (nucleoporin 58) are associated with androgen receptors in adrenal gland function or steroid hormone pathways, suggesting that they play roles in modulating androgen receptor activity and steroid hormone production [26,27]. An elevated expression level of *Kiss1* (Kisspeptin1) was also identified; its protein product is known to regulate adrenal steroidogenesis through modulation of the hypothalamic–pituitary–adrenal axis [28,29]. Furthermore, *H2-Ke6* (Hsd17b8, hydroxysteroid 17-beta dehydrogenase 8) participates in the conversion of less active steroid hormones to more active forms, such as converting estrone to estradiol [30,31].

**Figure 5:**
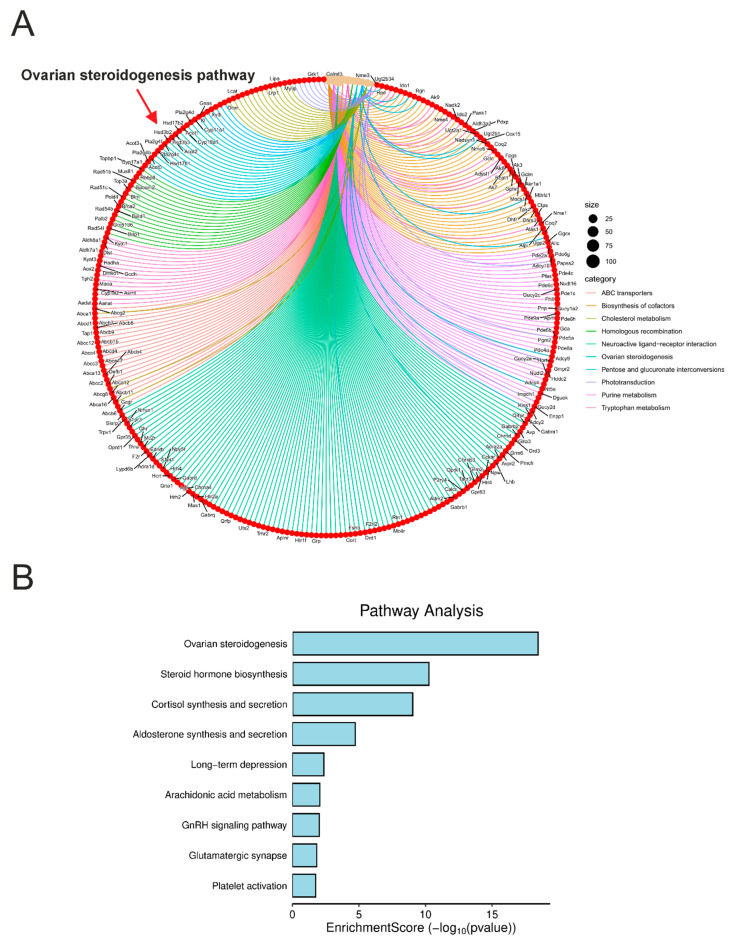
KEGG pathway enrichment analysis of differentially expressed genes in adrenal glands of *Acomys cahirinus*. (**A**) Cnet plot highlighting enriched pathways, including steroidogenesis. (**B**) Bar plot of enrichment scores (−log10 FDR) for the top biological pathways and processes, including the KEGG term ovarian steroidogenesis (which features the *STAR* gene).

We performed a gene ontology enrichment analysis on differentially expressed genes in *Acomys.* This analysis revealed significant enrichment of a KEGG pathway annotated as ovarian steroidogenesis (Figure 5A,B). It is important to note that this designation reflects the KEGG database nomenclature, where the pathway (map04913) includes enzymes involved in the production of sex steroids in the ovary. However, these enzymatic steps are largely shared between ovarian and adrenal steroidogenic processes. Therefore, the activation of this pathway in our data likely represents adrenal cortex activity related to androgen biosynthesis, rather than ovary-specific functions.

To explore this pathway further, we included *STAR*, a gene known to play a central role in steroidogenesis. This led to the identification of additional enriched metabolic routes associated with steroid hormone biosynthesis, as well as cortisol synthesis and secretion (Figure 5B). These findings provide indirect evidence for the existence of a functionally active adrenal steroidogenic pathway in *Acomys cahirinus*, potentially supporting both androgen and cortisol production.

A notable observation was the decreased expression of *Cyb5a* in *Acomys* relative to Balb/c. Additionally, due to the limitations of the preliminary *Acomys* genome annotation (based on liftover from the mouse genome), we currently lack information on the expression of *Sult2a1*, a key enzyme for adrenal sex steroid production in humans. The absence (or undetectable expression) of this gene in our dataset may suggest that *Acomys* utilizes alternative enzymes or metabolic routes for the synthesis and regulation of adrenal steroids.

**Figure 6:**
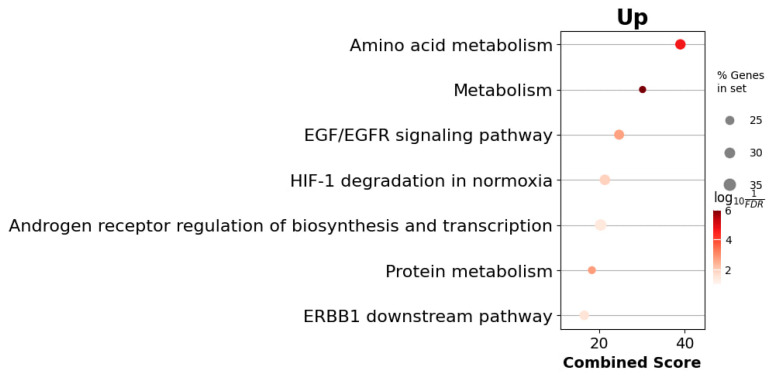
BioPlanet 2019 enrichment analysis of *Acomys cahirinus* adrenal gland DEGs.

Subsequently, we performed over-representation analysis (ORA) using the gseapy python package and the BioPlanet_2019 gene set collection. (Figure 6) This analysis revealed statistically significant enrichment of pathways and processes closely linked to adrenal gland function and hormone secretion. Specifically, enriched terms such as Metabolism, Amino acid metabolism and Protein metabolism highlight the regulatory role of adrenal hormones like cortisol in metabolic processes. The EGF/EGFR signaling pathway and ERBB1 downstream pathway suggest involvement in the proliferation and survival of adrenal cortical cells. Additionally, the term Androgen receptor regulation of biosynthesis and transcription reflects the adrenal glands’ role in androgen production and signaling, while HIF-1 degradation in normoxia points to the influence of hypoxic and stress conditions on adrenal hormone regulation.

**Figure 7:**
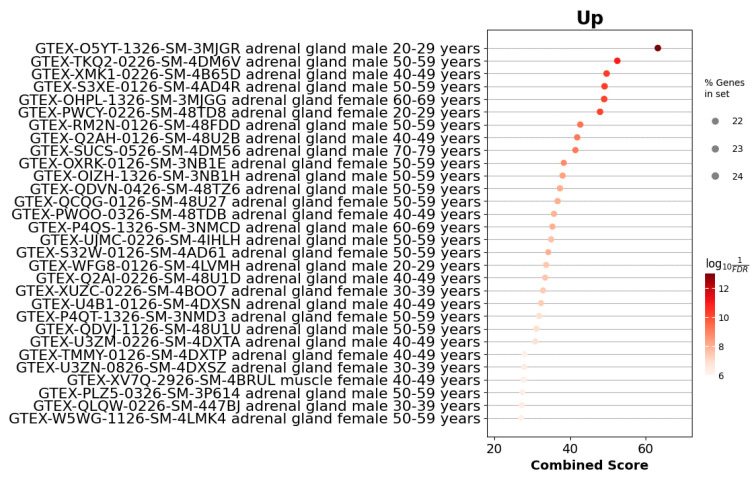
GTEx tissue enrichment analysis for upregulated genes in *Acomys cahirinus* adrenal glands. The dot plot shows samples from the GTEx Tissue Expression Up library ranked by combined enrichment score. Each point represents a GTEx sample annotated by tissue, sex, and age group. The dot size corresponds to the proportion of input DEGs found in the sample’s signature gene set (% Genes in set), and the color intensity reflects the statistical significance as −log_10_(FDR).

**Figure 8:**
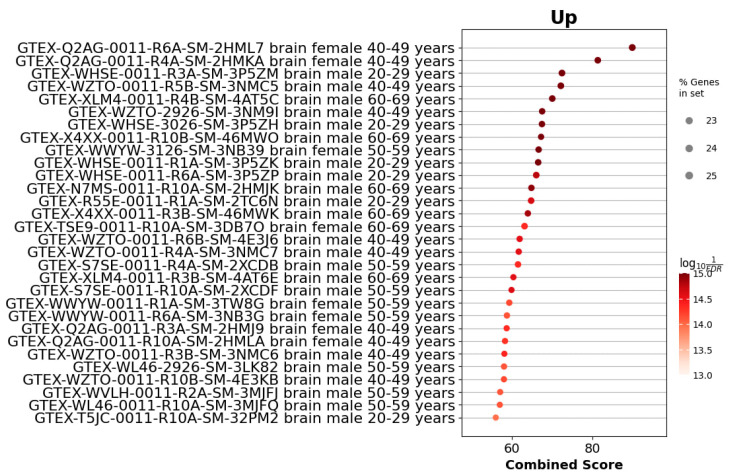
GTEx tissue enrichment analysis for upregulated genes in *Mus musculus* (Balb/c) adrenal glands. The dot plot shows samples from the GTEx Tissue Expression Up library ranked by combined enrichment score. Each point represents a GTEx sample annotated by tissue, sex, and age group. The dot size corresponds to the proportion of input DEGs found in the sample’s signature gene set (% Genes in set), and the color intensity reflects the statistical significance as −log_10_(FDR).

To assess the similarity of *Acomys* gene expression profiles to those of human tissues, we utilized the Enrirchr GTEx_Tissue_Expression_Up library, which contains gene sets enriched in specific human tissues. Our analysis revealed significant enrichment of gene signatures associated with human adrenal gland tissue, with both statistically significant *p*-values and high combined scores, suggesting a close resemblance of *Acomys* tissue specificity to that of the human adrenal gland (Figure 7). However, when we analyzed the differentially expressed genes in mouse samples, we observed a clear off-target shift toward gene expression signatures of a different tissue (Figure 8).

**Orthogonal Validation of RNA-seq Findings by qRT-PCR and CAGE-seq**

In order to validate the results of the bulk RNA-seq, two orthogonal approaches were employed: quantitative real-time PCR (qRT-PCR) and cap analysis of gene expression (CAGE).

**Figure 9:**
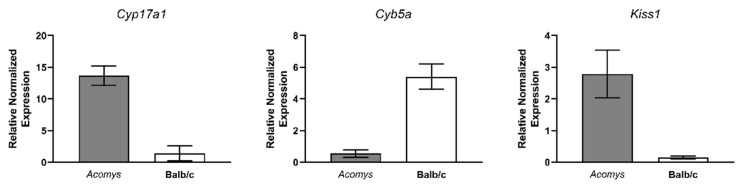
Quantitative real-time PCR validation of differential gene expression in adrenal glands of *Acomys cahirinus* and Balb/c mice. Relative expression levels of *Cyp17a1*, *Kiss1*, and *Cyb5a* genes. n = 5.

The qRT-PCR assay confirmed the differential expression of three high-abundance genes—*Cyp17a1* and *Kiss1* were upregulated, whereas *Cyb5a* was downregulated in *Acomys cahirinus* relative to Balb/c mice (Figure 9). For five transcripts (*Sult1e1, Hsd3b2, Defb18, Nupl1*, and *H2-Ke6*), qRT-PCR did not reproduce the direction of change detected by RNA-seq, likely owing to isoform-specific primer bias and differences in 5′- versus 3′-end coverage between the two methods. To obtain an independent read-out, we analyzed promoter activity with CAGE-seq, which corroborated the RNA-seq directionality for four of these genes.

A comparative analysis of the CAGE transcriptomes for the adrenal glands of *Acomys cahirinus* and Balb/c is presented in Supplementary Figure S2. CAGE sequencing further corroborated the transcriptional activity of these genes, demonstrating strong concordance between both methods for the majority of targets (Table 1). However, a notable exception was *Nupl1*, which exhibited discordant expression patterns between the RNA-seq and CAGE datasets. This discrepancy may be indicative of technical differences in transcript detection or potential post-transcriptional regulation of *Nupl1*, necessitating further investigation.

These validation experiments serve to enhance the reliability of our transcriptomic findings, while simultaneously underscoring the significance of orthogonal verification in the context of genomic studies.

## 4. Discussion

This study provides strong evidence for the potential of *Acomys cahirinus* as a valuable model for human adrenal gland research, offering several advantages over the conventional *Mus musculus* (Balb/c mouse) model. The findings demonstrate substantial structural and molecular similarities between the adrenal glands of *Acomys cahirinus* and humans, suggesting that this species may serve as a more relevant preclinical model for studying adrenal pathologies and for the development of targeted therapies.

One of the most striking observations in this study is the presence of a well-defined zona reticularis in the adrenal cortex of *Acomys cahirinus*, a feature that is absent in Balb/c mice but is a key characteristic of the human adrenal gland. This structural similarity suggests that *Acomys cahirinus* may more accurately recapitulate the functional organization of the human adrenal gland, potentially leading to more translatable research outcomes in studies of adrenal physiology and pathology.

The differential gene expression analysis revealed several genes involved in androgen synthesis and steroid hormone regulation that were upregulated in *Acomys cahirinus* compared to Balb/c mice. Notably, the increased expression of *Cyp17a1*, *Sult1e1*, and *Hsd3b2* in *Acomys cahirinus* suggests a more active steroidogenic pathway, particularly in androgen production. This finding is particularly relevant for studying androgen-related disorders, such as congenital adrenal hyperplasia or adrenal androgen-producing tumors, where *Acomys cahirinus* may provide a more accurate representation of human pathophysiology.

The gene ontology enrichment analysis further supports the relevance of *Acomys cahirinus* as a model for human adrenal function. The identification of pathways associated with ovarian steroidogenesis and steroid hormone biosynthesis as well as cortisol synthesis and secretion in *Acomys cahirinus* indicates the presence of functionally active metabolic pathways that contribute to the production of cortisol and androgens. This finding is particularly significant, as it suggests that *Acomys cahirinus* may be a suitable model for studying disorders of cortisol production, such as Cushing’s syndrome or adrenal insufficiency.

Over-representation analysis (BioPlanet-2019) highlighted a single pathway of particular interest—“androgen-receptor regulation of biosynthesis and transcription”. The enrichment of this module indicates that steroidogenic output in Acomys adrenals is coupled to active intra-adrenal androgen-receptor signaling. This finding dovetails with our gene-level data for *Cyp17a1*, *Kiss1*, and *H2-Ke6*, collectively supporting the notion that *Acomys cahirinus* reproduces human-like androgen regulation within the adrenal cortex.

Interestingly, the decreased expression of *Cyb5a* in *Acomys cahirinus* compared to Balb/c mice, along with the potential absence of expression of *Sult2a1*, suggests that this species may utilize alternative enzymes or metabolic routes for the synthesis and regulation of steroid hormones. This observation opens up new avenues for research into novel steroidogenic pathways and could potentially lead to the discovery of alternative therapeutic targets for adrenal disorders.

GTEx-based tissue-signature enrichment revealed that the *Acomys* adrenal gene set most closely aligns with the human adrenal cortex, whereas Balb/c DEGs shifted toward brain-associated profiles. This result extends earlier morphologic and single-gene evidence, providing a quantitative, genome-wide indication that *Acomys* captures human-like adrenal tissue specificity better than the standard mouse model. Such proximity is particularly valuable for preclinical studies where off-target transcriptional programs in *Mus musculus*—as seen here—might confound drug-response or gene-therapy read-outs. The data therefore strengthen the case for adopting *Acomys cahirinus* as a complementary model in translational adrenal research, especially for disorders in which precise regulation of human-type steroid networks is critical.

While these findings are promising, it is important to acknowledge the limitations of the study. The relatively small sample size and the focus on sexually mature male mice may limit the generalizability of the results. Future studies should aim to include larger sample sizes and investigate both genders across different developmental stages to provide a more comprehensive understanding of adrenal gland function in *Acomys cahirinus*.

Additionally, the use of bulk RNA sequencing in this study, while informative, does not capture the full complexity and heterogeneity of adrenal gland cell populations. To address this limitation, future research should incorporate single-cell RNA sequencing techniques, which would provide a more detailed transcriptomic profile of the *Acomys* adrenal glands and allow for the identification of cell type-specific gene expression patterns.

## 5. Limitations

This study has several limitations. First, only male animals were included. We chose males to minimize endocrine variability when establishing a cross-species baseline, because female *Acomys cahirinus* exhibit cyclical ovarian hormone fluctuations that can influence adrenal transcription [32]. Nevertheless, this design limits generalizability across sexes. Second, our analyses rely on bulk RNA-seq and promoter-level CAGE-seq, which do not resolve cell-type-specific programs. Single-cell and spatial transcriptomics will be required to assign zone- and cell-specific signals.

## 6. Conclusions

This study presents evidence for the potential of *Acomys cahirinus* as a valuable model for human adrenal gland research. The structural and molecular parallels between *Acomys* and human adrenal glands, particularly the presence of the zona reticularis and the expression of key steroidogenic genes, suggest that this species may offer significant advantages over conventional mouse models in the study of adrenal physiology and pathology.

Moving forward, it will be crucial to further validate and characterize the *Acomys cahirinus* model through comprehensive transcriptomic profiling at the single-cell level, as well as performing functional studies to confirm the physiological relevance of the observed gene expression patterns. Additionally, investigating the potential of *Acomys cahirinus* in modeling specific adrenal disorders, such as congenital adrenal hyperplasia or adrenocortical tumors, could provide valuable insights into disease mechanisms and facilitate the development of novel therapeutic approaches.

## Figures and Tables

**Table 1:** CAGE sequencing analysis of adrenal gland transcriptional activity in *Acomys cahirinus* and Balb/c mice.

Gene	Balb/c	*Acomys cahirinus*	DE
*Cyp17a1*	Not expressed	Active	-
*Sult1e1*	Not expressed	Active	-
*Hsd3b2*	Not expressed	Active	-
*Defb18*	Not expressed	Active	-
*Nupl1*	Active	Active	n/s (*p*-value > 0,08)
*Kiss1*	Not expressed	Active	-
*H2-Ke6*	Active	Active	*Acomys* > *Balb/c 1.8 times*; *p* = 2.6 × 10^−10^
*Cyb5a*	Active	Active	*Acomys* < *Balb/c 2.7 times*; \ *p* = 7.7 × 10^−9^

## Data Availability

The raw sequence data reported in this paper have been deposited in the Genome Sequence Archive (Genomics, Proteomics & Bioinformatics 2021) in the National Genomics Data Center (Nucleic Acids Res 2022), China National Center for Bioinformation/Beijing Institute of Genomics, Chinese Academy of Sciences (GSA: CRA015798); they are publicly accessible at https://ngdc.cncb.ac.cn/gsa (accessed on 11 September 2025).

## Supplementary Materials

The following supporting information can be downloaded at: https://www.mdpi.com/article/10.3390/cells14181431/s1. Table S1. Primer sequences used for RT-qPCR; Table S2. Differentially expressed genes; Figure S1. Volcano plot of all DEGs between Acomys and Balb/c adrenal glands; Figure S2. Comparative analysis of CAGE transcriptomes for adrenal glands of *Acomys cahirinus* and Balb/c.
