# Supplementary material for: Morphological and Transcriptomic Analyses of the Adrenal Gland in Acomys cahirinus: A Novel Model for Murine Adrenal Physiology
Source: Cells. 2025 Sep 12;14(18):1431. doi: 10.3390/cells14181431 (PMC12468302; doi:10.3390/cells14181431)
Supplement: Supplementary file 1 [file cells-14-01431-s001.zip › Supplementary Figure S1.pdf]

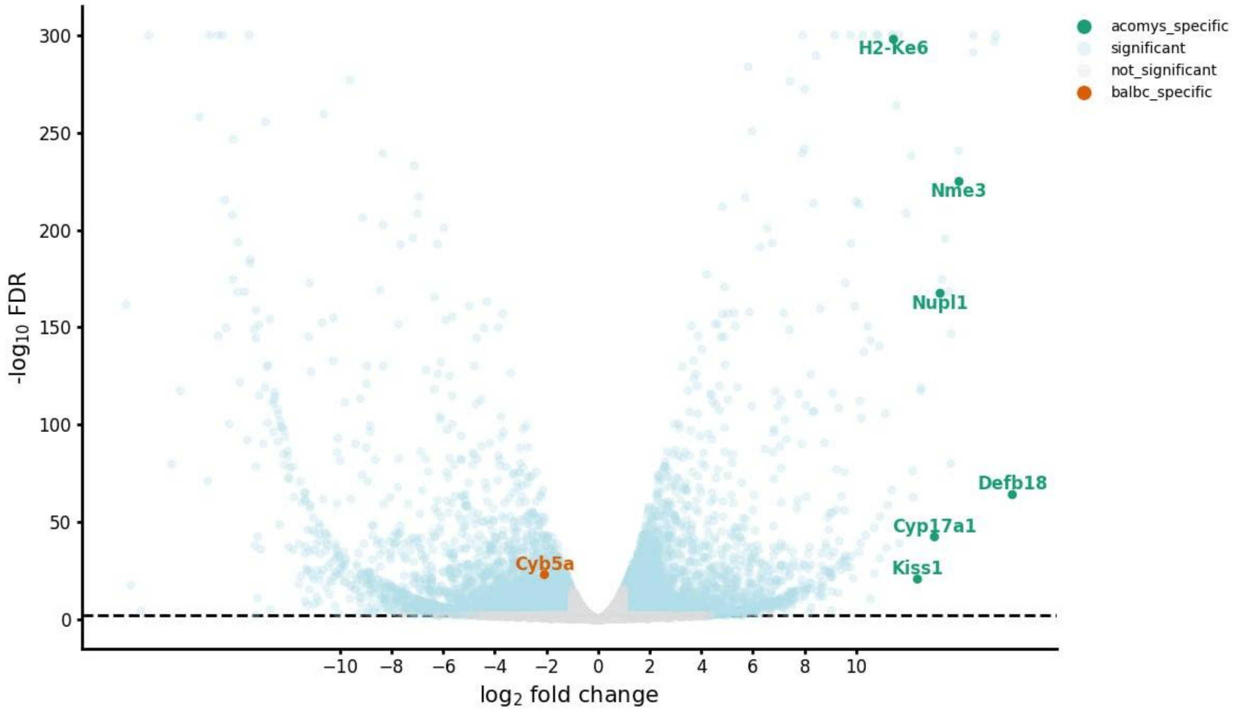

Figure S2. Volcano plot of differentially expressed genes (DEGs) in *Acomys* versus Balb/c adrenal glands.

Dots represent individual genes (log_2 FC vs $-log_{10}$ FDR). Green = *Acomys*-specific up-regulation; orange = Balb/c-specific; light blue = significant; grey = not significant. Key DEGs are labelled.
