# Supplementary material for: Morphological and Transcriptomic Analyses of the Adrenal Gland in Acomys cahirinus: A Novel Model for Murine Adrenal Physiology
Source: Cells. 2025 Sep 12;14(18):1431. doi: 10.3390/cells14181431 (PMC12468302; doi:10.3390/cells14181431)
Supplement: Supplementary file 1 [file cells-14-01431-s001.zip › Supplementary Figure S2.pdf]

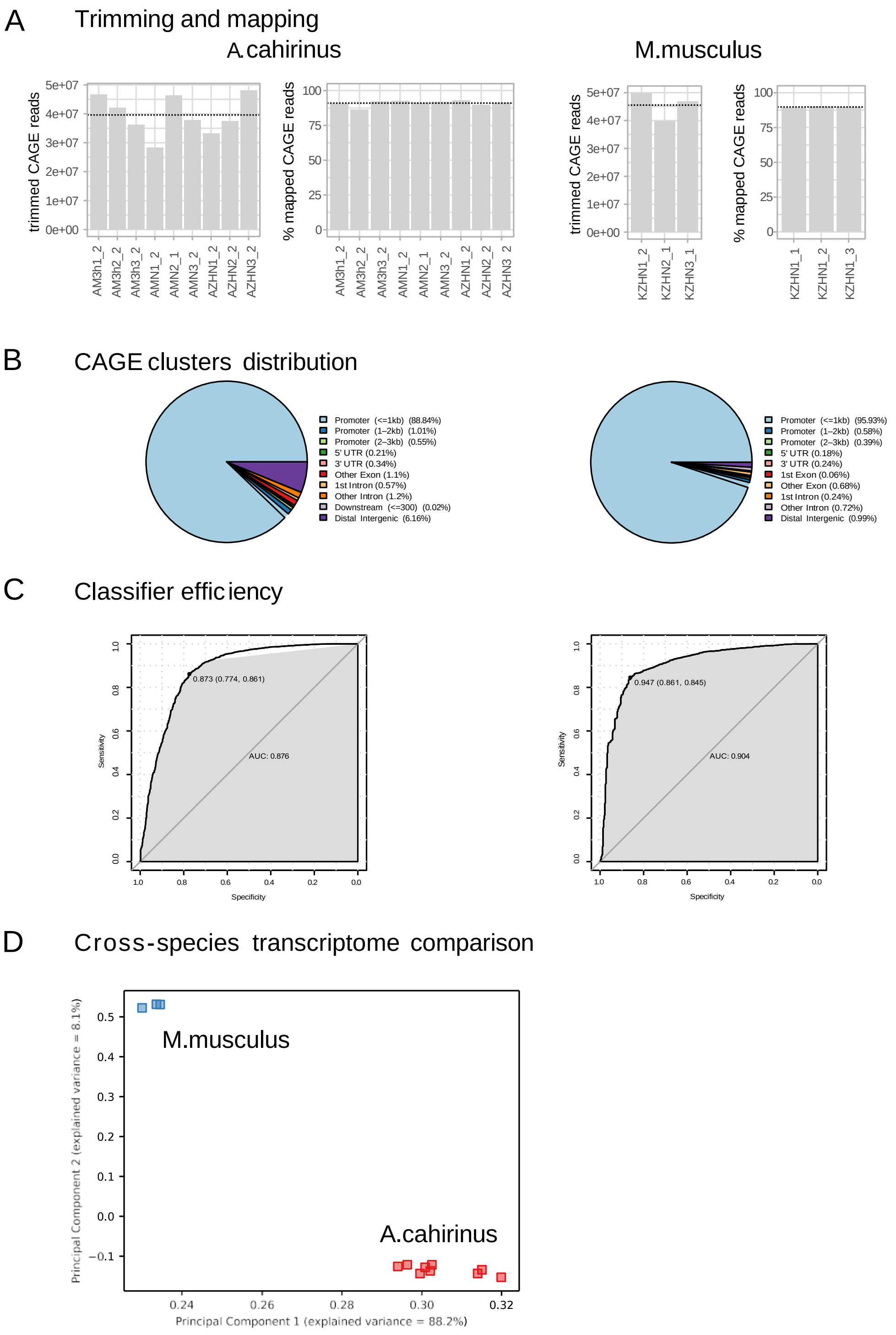

Figure S2. Comparative analysis of CAGE transcriptomes for adrenal glands of *Acomys cahirinus* and Balb/c. A. Sample sizes after trimming and genome mapping ratio. B. Genomic distribution of CAGE clusters for two species. C. Promoter classifier efficiency for two species. D. Cross - species transcriptome comparison with PCA. Counts of CAGE clusters located in known promoter regions or classified as promoter were used for cumulative expression estimation for each ortholog.
