# Supplementary material for: Morphological and Transcriptomic Analyses of the Adrenal Gland in Acomys cahirinus: A Novel Model for Murine Adrenal Physiology
Source: Cells. 2025 Sep 12;14(18):1431. doi: 10.3390/cells14181431 (PMC12468302; doi:10.3390/cells14181431)
Supplement: Supplementary file 1 [file cells-14-01431-s001.zip › Supplementary Table S1.pdf]

Table S1. Primer sequences used for RT-qPCR

**Organism**	**Targeted gene**	**Primer name**	**Primer sequence**
Mouse	Cyp17a1	Cyp17a1-M F	GACATATTCCCGTGGTTGAAGATTT
Mouse	Cyp17a1	Cyp17a1-M R	GTGTCCTTCGGGATGGCAAA
Mouse	Sult1e1	Sult1e1-M F	TGCAAGGGCAAGTTCCGTAT
Mouse	Sult1e1	Sult1e1-M R	GACACAAGGAAGTGGCTCAGA
Mouse	Hsd3b2	Hsd3b2-M F	TGGAGCAGGAGGGTTTTTGG
Mouse	Hsd3b2	Hsd3b2-M R	CCTCCAATAAGTTCTGGGTACCTTT
Mouse	Nupl1	Nupl1-M F	CACAGCTCCTGCTGACTACTT
Mouse	Nupl1	Nupl1-M R	CTGCCAAAGCCTGCACTAAG
Mouse	H2-Ke6	H2-Ke6-M F	GCATCATTGGAAAGGTGGGGA
Mouse	H2-Ke6	H2-Ke6-M R	TAGCCTGGGAACTTAGCGTC
Mouse	Kiss1	Kiss1-M F	CAGGACCCAGTCAAGTCCCT
Mouse	Kiss1	Kiss1-M R	CCTGCCTTGGCCTCTACAAT
Mouse	Defb18	Defb18-M F	CTGTGGTCCTTCTGCTCCTCAG
Mouse	Defb18	Defb18-M R	AGGGCTCAGTGTGACAGTAGTT
Mouse	Cyb5a	Cyb5a-M F	GCCAAGCCTTCGGATACTCT
Mouse	Cyb5a	Cyb5a-M R	CGTTCTCAGGGGGAGGTTTC
Mouse	GAPDH	GAPDH-M F	TCAGGAGAGTGTTTCCTCGTC
Mouse	GAPDH	GAPDH-M R	GTTGCTGTTGAAGTCGCAGG
Acomys	Cyp17a1	Cyp17a1-A F	TTCCTCATCCATGGCCAACC
Acomys	Cyp17a1	Cyp17a1-A R	CAAGGTTAGTGGGCAGGAGG
Acomys	Sult1e1	Sult1e1-A F	CATGGGCCTGGAACAGTGAA
Acomys	Sult1e1	Sult1e1-A R	GAAGAAGATCGGGTGGCAGG

Acomys	Hsd3b2	Hsd3b2-A F	TGGCGAGGAGGAGAAGATGA
Acomys	Hsd3b2	Hsd3b2-A R	AAGGTCTAGGTCTGGCCCAA
Acomys	Nupl1	Nupl1-A F	GGAGTAGTGGGTTGTCCTGC
Acomys	Nupl1	Nupl1-A R	GGAATTGGGGGTCTTCAGGG
Acomys	H2-Ke6	H2-Ke6-A F	GGGTCAGCATCCACTCTTCC
Acomys	H2-Ke6	H2-Ke6-A R	ACCCACTTCTGTCCCCTTCA
Acomys	Kiss1	Kiss1-A F	ACCTCCCCTACATCCCCTTC
Acomys	Kiss1	Kiss1-A R	GGCTAACAGACCCCTGAACC
Acomys	Defb18	Defb18-A F	AGGGGGAAAGGAGTGGTTGT
Acomys	Defb18	Defb18-A R	TTGCATACACCACGGACCAG
Acomys	Cyb5a	Cyb5a-A F	GGCTCACTGTTCCGAGATGG
Acomys	Cyb5a	Cyb5a-A R	GGTTCGGACATCACTCCCAG
Acomys	GAPDH	GAPDH-A F	GTTTGTGATGGGCGTGAACC
Acomys	GAPDH	GAPDH-A R	ATGCTGCTGGAGATGACCCT
Mouse+*Acomys*	ACTB	ACTB-AM F	CGCAGCCACTGTCGAGTC
Mouse+*Acomys*	ACTB	ACTB-AM R	CCACAGGATTCCATACCCAAGA
